# Comparison of Whole Genome (wg-) and Core Genome (cg-) MLST (BioNumerics^TM^) Versus SNP Variant Calling for Epidemiological Investigation of *Pseudomonas aeruginosa*

**DOI:** 10.3389/fmicb.2020.01729

**Published:** 2020-07-22

**Authors:** Dominique S. Blanc, Bárbara Magalhães, Isabelle Koenig, Laurence Senn, Bruno Grandbastien

**Affiliations:** Service of Hospital Preventive Medicine, Lausanne University Hospital, University of Lausanne, Lausanne, Switzerland

**Keywords:** whole genome sequencing, *Pseudomonas aeruginosa*, molecular typing, wgMLST, cgMLST, evaluation

## Abstract

Whole genome sequencing (WGS) is increasingly used for epidemiological investigations of pathogens. While SNP variant calling is currently considered as the most suitable method, the choice of a representative reference genome and the isolate dependency of results limit standardization and affect resolution in an unknown manner. Whole or core genome Multi Locus Sequence Typing (wg-, cg-MLST) represents an attractive alternative. Here, we assess the accuracy of wg- and cg-MLST by comparing results of four *Pseudomonas aeruginosa* datasets for which epidemiological and genomic data were previously described. Three datasets included 155 isolates from three different sequence types (ST) of *P. aeruginosa* collected in our ICUs over a 5-year period. The fourth dataset consisted of 10 isolates from an investigation of *P. aeruginosa* contaminated hand soap. All isolates were previously analyzed by a core SNP approach. In this study, wg- and cg-MLST were performed in BioNumerics^TM^ using a scheme developed by Applied-Maths. Correlation between SNP calling and wg- or cg-MLST results were evaluated by calculating linear regressions and their coefficient of correlations (*R*^2^) between the number of SNPs and the number of allele differences in pairwise comparison of isolates. The number of SNPs and allele difference between isolates with close epidemiological linkage varies between 0–26 and 0–13, respectively. When compared to core-SNP calling, a higher coefficient of correlation was obtained with cgMLST (*R*^2^ of 0.92–0.99) than with wgMLST (0.78–0.99). In one dataset, a putative homologous recombination of a large DNA fragment (202 loci) was identified among these isolates, affecting its phylogeny, but with no impact on the epidemiological analysis of outbreak isolates. In conclusion, we showed that the *P. aeruginosa* wgMLST scheme in BioNumerics^TM^ is as discriminatory as the core-SNP calling approach and apparently useful for outbreak investigations. We also showed that epidemiological linked isolates showed less than 26 SNPs or 13 allele differences. These are important figures for the distinction between outbreak and non-outbreak isolates when interpreting WGS results. However, as *P. aeruginosa* is highly recombinant, a cgMLST approach is preferable and caution should be addressed to possible recombination of large DNA fragments.

## Introduction

*Pseudomonas aeruginosa* is considered one of the main Gram-negative bacterial species causing health-care associated infections (Gellatly and Hancock, 2013). In the hospital setting, *P. aeruginosa* is widely present in the environment and can be retrieved from different sources, such as respiratory therapy equipment, antiseptics, soap, sinks, and hydrotherapy pools (Morrison and Wenzel, 1984). This opportunistic pathogen was also found to be part of the endogenous microbiota of up to 24% of hospitalized patients (Morrison and Wenzel, 1984). Patients with compromised host defense mechanisms, such as neutropenia, severe burns, or cystic fibrosis, are particularly affected by infections which lead to high morbidity and mortality (Gellatly and Hancock, 2013). *P. aeruginosa* has been previously described as the second most common organism responsible for infections acquired in intensive care units (ICUs) (Vincent et al., 1995). For these reasons, active surveillance of nosocomial infection and outbreak detection is of paramount importance. However, as *P. aeruginosa* possesses a very complex ecology, only powerful typing methods can give insight on the relatedness of strains and, consequently, on the routes of colonization and/or infection (Maiden et al., 2013).

One of the key postulates of molecular epidemiology is that phylogeny approximates epidemiology: isolates that are phylogenetically closely related have a common ancestor and thus derive from a chain of transmission (Struelens, 1996; van Belkum et al., 2007). Similarly, if pathogens retrieved from the environment are phylogenetically related to clinical isolates, a causal relationship between the two is likely (Besser et al., 2018). Higher is the discriminatory power of the typing method, stronger will be this postulate. In recent years, whole genome sequencing (WGS) has shown to provide a much higher discriminatory power in comparison to other technologies such as pulsed field gel electrophoresis (PFGE) or multi-locus sequencing typing (MLST) (Gilchrist et al., 2015; Allard, 2016).

Several approaches are used to analyse WGS data for epidemiological and infection control purposes. A popular approach is to construct phylogenies based on Single Nucleotide Polymorphism (SNP) variant calling, which identifies single nucleotide differences between isolates to a single reference genome. The requirement for such a reference genome is a limitation, since the reference should be closely related to the genome of the studied isolates to identify true phylogenetically informative SNPs (Besser et al., 2018; Parcell et al., 2018). Since a reference genome might not always be available, results are dependent on the chosen genome and the collection of isolates, limiting standardization and influencing the resolution in an unknown fashion (Pightling et al., 2014). An alternative approach is a gene-by-gene typing method, indexing core or accessory gene variation due to mutations or recombination events (Maiden et al., 2013; Pearce et al., 2018). This can be considered as an extended MLST scheme. The core genome (cg) MLST includes loci that are present in all isolates of a given population or a subset thereof (Maiden et al., 2013), whereas whole genome (wg) MLST adds a selection of accessory loci as well. An advantage of this approach is that, as with MLST, loci used in the schemes are readily maintained and shared among laboratories using the same or similar online databases (Maiden et al., 2013). Previous studies within other bacterial species have shown SNP analyses and cgMLST to be largely congruent (Kohl et al., 2014; Lee et al., 2014; Pearce et al., 2018).

Here, we aimed at comparing results of wg- and cg-MLST using Bionumerics schemes to results previously obtained by SNP analyses during outbreak investigation of *P. aeruginosa.* Four published epidemiological investigations performed in our hospital were used for this purpose. We also aimed at describing the number of allele and SNP differences observed among outbreak-related isolates using epidemiological information. This information will provide a useful and consistent starting point for interpreting WGS results for *P. aeruginosa* in the hospital setting.

## Materials and Methods

### Description of the Four Datasets

The first three datasets included 155 isolates from the three predominant *P. aeruginosa* MLST sequence types (ST) (ST17, 253 and 1076) collected in our ICUs over a 5-year surveillance period (2010–2014) (Magalhães et al., 2020) (Table 1). In summary, during this period, all consecutive patients hospitalized in the ICU with a clinical sample growing *P. aeruginosa* at any site were considered. In 2012, the ICU environment was investigated for the presence of *P. aeruginosa*. Tap water samples and environmental swabs obtained from taps and sink traps of all patient’s rooms were analyzed. Thereafter, sink traps were investigated twice a year. All isolates were analyzed with molecular typing and the three predominant MLST STs were further investigated with WGS. The first dataset, ST1076, includes isolates from an outbreak occurring in the burn unit (Tissot et al., 2016). With one exception, epidemiological links were found between these patients, either through environmental contamination or through patient-to-patient transmission. Regarding the second dataset, ST17, epidemiological links were found between five patients hospitalized in the burn unit in 2010. For the other 16 patients, no epidemiological link was suspected, as patients were not hospitalized in the same ICU during overlapping periods and had no link with environmental samples. Within the third dataset, ST253, epidemiological links were identified only between two patients hospitalized in the same ICU and an environmental sample retrieved from a sink trap. The remaining patients were dispersed throughout the six ICUs during the study period, suggesting they were not involved in an outbreak. The fourth dataset consisted of 10 isolates of ST155 from an epidemiological investigation following the contamination of hand soap with *P. aeruginosa* (Blanc et al., 2016) (Table 1).

**TABLE 1:** Description of the four datasets.

**Dataset**	**Investigation**	**No. of isolates**	**MLST ST**	**References**	**Read files accession number**
1	Outbreak in ICU	74	1076	Magalhães et al., 2020	PRJNA503802
2	ICU surveillance	50	17	Magalhães et al., 2020	PRJNA503802
3	ICU surveillance	31	253	Magalhães et al., 2020	PRJNA503802
4	Hand soap contamination	10	155	Blanc et al., 2016	PRJEB36387

All datasets were previously analyzed with a SNP variant calling approach with exclusion of putative recombinant segments (Tissot et al., 2016; Magalhães et al., 2020). The read data of the first three datasets have been deposited at the Sequence Read Archive (SRA)^1^ under the accession project number PRJNA503802. The read data of the fourth dataset have been deposited with the European Nucleotide Archives (ENA)^2^ under the accession study number PRJEB36387.

### wgMLST Analysis

Wg- and cg-MLST was performed in BioNumerics^TM^ (version 7.6.3, created by bioMérieux (Applied Maths NV, St Martens Latem, Belgium), available at http://www.applied-maths.com) with default parameters using a scheme containing 15.136 loci and the 7 public MLST loci. In brief, allele calling can be performed using either of two processes: (i) assembly free allele calling is done directly on the reads (if available) by comparing kmer frequency tables of the reads to kmer frequency tables of all known alleles (using a kmer size of 35; only kmers found in 3 or more reads, with at least 1 forward and 1 reverse, are included), and (ii) assembly based allele calling. For this latter process, first a *de novo* assembly is performed using SPAdes 3.7.1 followed by a mismatch correction with bwa-mem, removal of contigs below 300 bp, and a consensus calling on the output (single base threshold 75%, double base threshold 85%, triple base threshold 95% and gap threshold of 50%). Positions in the assembly covered by less than 3 reads, 1 forward or 1 reverse read were replaced by N. Briefly, reference alleles were blasted against the resulting consensus with megablast, using an identity threshold of 85%. Blast hits were compared with known high quality alleles. If no match was found in this set and the hit contains a valid start and stop within 18 bp of the start and stop of the reference and no internal stop, the allele is assigned a new ID. Finally, a consensus allele call is performed with both the assembly free and the *de novo* assembly based calling, results found with both algorithms or with only one algorithm are maintained, discrepant results are removed. cgMLST consisted in the selection of loci that were present in >90% of all isolates in the analyzed dataset (see Supplementary Material).

Clustering was performed using the categorical-difference coefficient and the tree was built using the UPGMA algorithm. The allele pairwise distance matrix was exported for comparison with the SNP pairwise distance matrix.

### Concordance Between SNPs Calling Variant and wg- and cg-MLST

The concordance between SNP and wg- or cg-MLST was assessed by calculating the correlation between the pairwise similarities between isolates obtained by each method. Each isolate was compared to the others with the SNPs-based approach resulting in a SNPs pairwise distance matrix. Similarly, a matrix with allele pairwise differences was calculated with the gene-by-gene approach. The concordance between the two matrices was plotted on a graph and a regression line was calculated including a coefficient of correlation (*R*^2^) using Excel.

## Results

**FIGURE 1:**
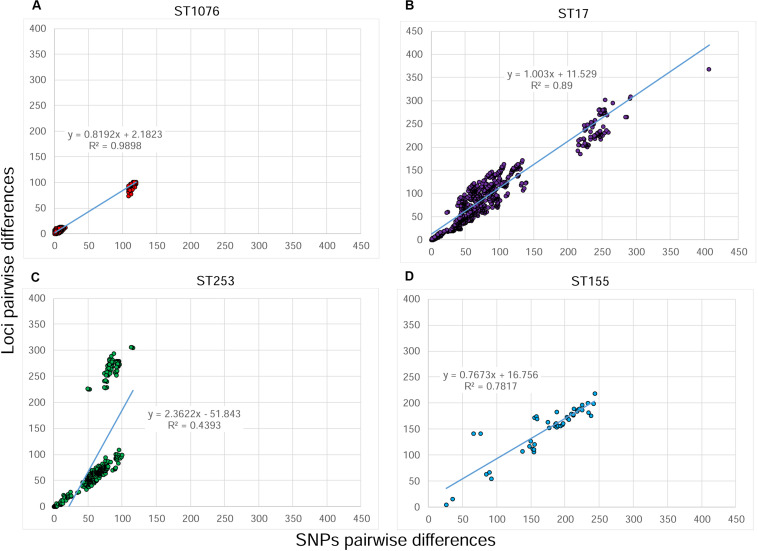
Correlation between SNPs and alleles pairwise distances of four sets of *Pseudomonas aeruginosa* isolates analyzed by whole genome sequencing. The number of SNP differences were obtained by a SNP variant calling analysis, whereas the number of allele differences originated from wgMLST analysis. **(A)** ST1076 dataset. **(B)** ST253 dataset, **(C)** ST17 dataset. **(D)** ST155 dataset. The regression line, its equation and the coefficient of determination (*R*^2^) are indicated on each plot.

The ST1076 dataset was composed of 74 isolates from a well characterized outbreak (Tissot et al., 2016), during which the first patient (two isolates) was excluded based on genotyping results (Tissot et al., 2016; Magalhães et al., 2020). wgMLST identified 6066 loci that were present in at least one of these 74 isolates. Among these, 153 loci were variable (Supplementary Table S1). The identification of genetically highly related isolates was concordant with previously acquired epidemiological information: differences of 0–16 SNPs and 0–12 alleles were observed among outbreak’ isolates, whereas differences of 108–120 SNPs and 74–101 alleles were observed between these isolates and the two non-outbreak isolates of patient #1. The linear regression between the pairwise SNP distances and the pairwise allele differences is shown in Figure 1A and a high coefficient of correlation was observed (*R*^2^ = 0.99). The clustering obtained by the SNP variant calling and the wgMLST approaches were highly similar (Supplementary Figure S1).

**FIGURE 2:**
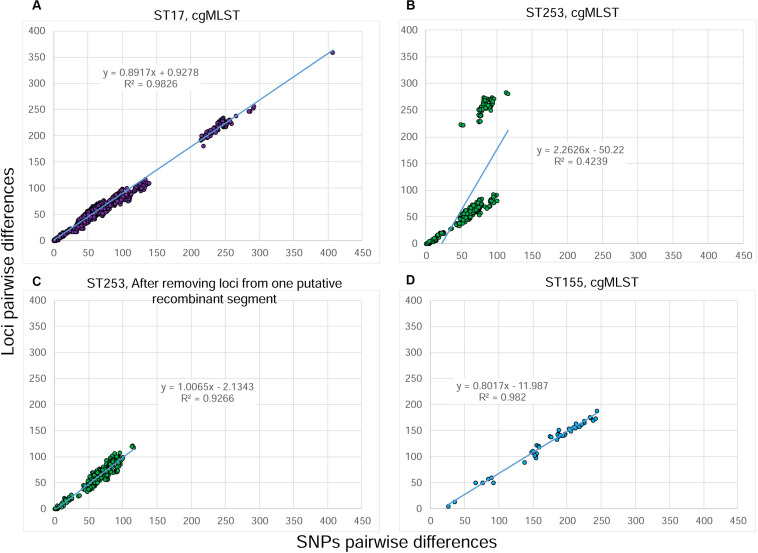
Correlation between SNPs and pairwise distances of *Pseudomonas aeruginosa* isolates analyzed by whole genome sequencing. The number of SNP differences were obtained by a SNP variant calling analysis, whereas the number of allele differences originated from wgMLST or cgMLST analysis. **(A)** ST17 dataset analyzed by a “relative” cgMLST analysis. **(B)** ST253 dataset analyzed by a “relative” cgMLST analysis. **(C)** ST253 dataset analyzed by wgMLST after removing loci from a segment of putative homologous recombination. **(D)**. ST155 dataset analyzed by a “relative” cgMLST analysis. The regression line, its equation and the coefficient of determination (*R*^2^) are indicated on each plot.

The ST17 dataset is composed of 50 isolates that originated from the previously mentioned surveillance in the ICU over a 5 year-period. Epidemiological links were found only among five patients hospitalized in the same ICU over a short period. For the other 16 patients, no epidemiological link was suspected. wgMLST identified 1114/6678 variable loci among these 50 isolates (Supplementary Table S2). Differences of 0–13 SNPs and 0–17 alleles were observed among epidemiologically linked isolates. The clustering obtained by the SNP variant calling and the wgMLST approaches were highly similar (Supplementary Figure S2). The correlation between SNPs and alleles pairwise differences (Figure 1B) was lower than in the first dataset (*R*^2^ = 0.89). One explanation for the low correlation is that each distance between two isolates obtained with the wgMLST analysis is based on the pairwise comparison of all loci present in both isolates (core and accessory loci), whereas the SNP analysis takes into consideration only positions where a nucleotide is present in all isolates of the dataset (core genome). This means that a larger percentage of the genome is considered in the wgMLST analysis vs. the core-SNP approach. To test this hypothesis, we performed a cgMLST analysis in Bionumerics^TM^ considering only loci with alleles present in at least 90% of isolates of the dataset (see Supplementary Material for more details). This cgMLST identified 838/5932 variable loci (Supplementary Table S2). The newly obtained distance matrix was compared to the original SNP matrix (Figure 2A) and the plot shows a much better correlation (*R*^2^ = 0.983), confirming that the lower correlation was due to the absence of loci in some isolates of the dataset.

The ST253 dataset was composed of 31 isolates also originating from surveillance in the ICU. wgMLST identified 667/7077 variable loci (Supplementary Table S3). Epidemiological links were identified only between two hospitalized patients and an environmental sample; differences of 0–1 SNP and zero alleles were observed between the isolates. The remaining patients were dispersed throughout the different ICUs during the study period, suggesting there was no transmission. The linear regression between SNPs and alleles pairwise differences shows a low correlation (*R*^2^ = 0.439) due to the presence of two clouds of dots; one showing a possible good correlation between the two variables and a second suggesting a higher number of alleles compared to the number of SNPs (Figure 1C). The clustering obtained by the SNP variant calling and the wgMLST approaches were similar, except a group of three environmental isolates that clustered distantly to the others in the wgMLST tree (Supplementary Figure S3). Similarly to the second dataset, a cgMLST was performed and 545/5795 variable loci were identified. The newly obtained distance matrix was compared to the original SNP matrix and the plot shows the persistence of the second cloud (Figure 2B). We hypothesized the occurrence of a recombinant segment that was included in the wgMLST analysis, but excluded in the core SNP variant calling analysis. To test this hypothesis, we visually inspected the allele value of the dataset in order to identify polymorph adjacent loci suggesting a homologous recombination. Such a segment of 196 loci was identified (PSEUAE03725 to PSEUAE03927, Supplementary Table S3). These loci were unselected from the cgMLST scheme and a new comparison performed showing 358/5599 variable loci. The distance matrix was compared to the original SNP matrix and the results were plotted in Figure 2C. The second cloud was no longer present and a high level of correlation was observed between SNPs and alleles pairwise distances (*R*^2^ = 0.927). These loci are found on the reference genome N15-01092 (accession number CP012901) between 4.75 and 5 Mbp (Applied Maths, personal communication).

Lastly, the ST155 dataset is composed of 10 isolates from an investigation of contaminated hand soap with *P. aeruginosa* (Blanc et al., 2016). Two isolates originated from the soap and the remaining isolates from patients. wgMLST identified 656/6920 variable loci (Supplementary Table S4). A difference of 26 SNPs and 5 alleles was observed between the two soap isolates. The correlation pairwise differences between SNPs and alleles (Figure 1D) was good (*R*^2^ = 0.782) and the clustering very similar (Supplementary Figure S4). Nevertheless, we similarly performed a cgMLST analysis showing 407/5755 variable loci and the correlation between these loci and the SNP calling was also found to be much better (*R*^2^ = 0.982, Figure 2D).

## Discussion

As demonstrated in this study, both SNP calling and wgMLST accurately distinguishes outbreak-related isolates from non-outbreak isolates. The identification of genetically closely related isolates using wgMLST was highly concordant with epidemiological data and SNP calling results for all outbreaks in this study. Within each outbreak, very few genetic differences, i.e., < 26 SNVs and <17 wgMLST alleles, were observed between epidemiologically related isolates. These figures provide useful and consistent starting point for interpreting WGS results for *P. aeruginosa* in the hospital setting.

In panmictic species such as *P. aeruginosa*, recombination is frequent and affects the phylogeny as it yields mosaic genes that have different evolutionary histories. One of the advantage of using the allele as the central unit in MLST schemes is the reduction of the effect of recombination due to accessory genes or homologous recombination of small DNA segments (usually the size of one or only a few genes). Both MLST and wgMLST analyses consider a recombination as a single mutational even, whereas the SNP calling analysis considers each SNP in the recombinant segment as an additional mutation. However, as the wgMLST scheme includes accessory genes, the presence/absence of these genes might have an impact on the resulting phylogeny. This is shown in our study using three datasets of *P. aeruginosa* isolates where the correlation between the number of core-SNP differences and the number of allele differences was higher for a cgMLST rather than a wgMLST scheme. We defined a cgMLST consisting of loci with alleles present in at least 90% of isolates of the dataset. As expected, this was accompanied with a loss of discrimination as the loss of variable loci varies from 2 to 38% between wgMLST and cgMLST; but had no influence on the number of allele differences among our strongly clonal outbreak isolates (Supplementary Table S1). These results show the importance of using a cgMLST scheme to evaluate the effect of recombination on the genetic similarity between isolates. Such schemes have been proposed, but none of the studies addressed a comparison with a SNP calling approach or the effect of recombination (Mellmann et al., 2016; de Sales et al., 2020; Perez-Vazquez et al., 2020; Royer et al., 2020; Stanton et al., 2020).

Homologous recombination due to large DNA segments will not be considered as a single genomic event with the wg- or cgMLST approach as it involves several loci. This will have an impact on the phylogeny, as it was observed in isolates of the lineage ST253. If such an event would occurred during an outbreak, wg- or cgMLST analysis will show two groups of isolates differentiated by a high number of loci, suggesting a multi strains outbreak or the rejection of some isolates from the outbreak. In our study, all isolates with epidemiological links showed less than 26 SNPs or 13 allele differences, suggesting that recombination did not took place during the short evolution of *P. aeruginosa* isolates. Nevertheless, it is important to assess whether such recombination events occurred within outbreak isolates (Parcell et al., 2018).

In conclusion, we showed with four *P. aeruginosa* datasets that epidemiologically linked isolates were genetically highly related with both a core-SNPs calling approach and the wgMLST approach in BioNumerics. We also showed that epidemiological linked isolates showed less than 26 SNPs or 13 allele differences. These are important figures for the distinction between clonal and non-clonal isolates when interpreting WGS results in outbreak investigation. As *P. aeruginosa* has an epidemic-panmicitic population structure, a cgMLST approach was found to be more adapted than wgMLST, as it limits the effect of the presence/absence of accessory genes or homologous recombination of small DNA segment. However, homologous recombination of large DNA segments can still affect the phylogeny obtained with cgMLST analyses. Despite such an event was not observed within outbreak isolates of our datasets, it is important to be cautious and to use protocols that enable their recognition.

## Data Availability Statement

Publicly available datasets were analyzed in this study. This data can be found here in the NCBI under accession number PRJNA503802 and in the European Nucleotide Archives (ENA) under accession number PRJEB36387.

## Author Contributions

DB was the principal investigator and wrote the manuscript. LS and BG were co-investigators and reviewed the manuscript. BM and IK performed the analysis of some of the data. All authors contributed to the article and approved the submitted version.

## Conflict of Interest

The authors declare that the research was conducted in the absence of any commercial or financial relationships that could be construed as a potential conflict of interest.

## Footnotes

1 https://www.ncbi.nlm.nih.gov/sra

2 www.ebi.ac.uk/ena

## Supplementary Material

FIGURE S1 SNP and wgMLST similarity trees of ST1076 isolates.

FIGURE S2 SNP and wgMLST similarity trees of ST253 isolates.

FIGURE S3 SNP and wgMLST similarity trees of ST17 isolates.

FIGURE S4 SNP and wgMLST similarity trees of ST155 isolates.

TABLE S1 wg- and cgMLST loci and alleles of clusters of ST1076 isolates. The locus tag corresponds to NCBI nomenclature. A value of “-1” means the absence of an allele at this locus.

TABLE S2 wg- and cgMLST loci and alleles of clusters of ST17 isolates. The locus tag corresponds to NCBI nomenclature. A value of “-1” means the absence of an allele at this locus.

TABLE S3 wg- and cgMLST loci and alleles of clusters of ST253 isolates. The locus tag corresponds to NCBI nomenclature. A value of “-1” means the absence of an allele at this locus.

TABLE S4 wg- and cgMLST loci and alleles of clusters of ST155 isolates. The locus tag corresponds to NCBI nomenclature. A value of “-1” means the absence of an allele at this locus.

TABLE S5 Step by step procedure to perform a core genome MLST on a set of isolates in BioNumeric^TM^ version 7.6.3.

## References

[B1] Allard M. W. (2016). The future of whole-genome sequencing for public health and the clinic. *J. Clin. Microbiol.* 54 1946–1948. 10.1128/jcm.01082-16 27307454 PMC4963481

[B2] Besser J. Carleton H. A. Gerner-Smidt P. Lindsey R. L. Trees E. (2018). Next-generation sequencing technologies and their application to the study and control of bacterial infections. *Clin. Microbiol. Infect.* 24 335–341. 10.1016/j.cmi.2017.10.013 29074157 PMC5857210

[B3] Blanc D. S. Magalhaes B. G. Abdelbary M. Prod’hom G. Greub G. Wasserfallen J. B. (2016). Hand soap contamination by *Pseudomonas aeruginosa* in a tertiary care hospital: no evidence of impact on patients. *J. Hosp. Infect.* 93 63–67. 10.1016/j.jhin.2016.02.010 27021398

[B4] de Sales R. O. Migliorini L. B. Puga R. Kocsis B. Severino P. (2020). A core genome multilocus sequence typing scheme for *Pseudomonas aeruginosa*. *Front. Microbiol.* 11:1049. 10.3389/fmicb.2020.01049 32528447 PMC7264379

[B5] Gellatly S. L. Hancock R. E. (2013). *Pseudomonas aeruginosa*: new insights into pathogenesis and host defenses. *Pathog. Dis.* 67 159–173. 10.1111/2049-632x.12033 23620179

[B6] Gilchrist C. A. Turner S. D. Riley M. F. Petri W. A. Jr. Hewlett E. L. (2015). Whole-genome sequencing in outbreak analysis. *Clin. Microbiol. Rev.* 28 541–563. 25876885 10.1128/CMR.00075-13 PMC4399107

[B7] Kohl T. A. Diel R. Harmsen D. Rothganger J. Walter K. M. Merker M. (2014). Whole-genome-based *Mycobacterium tuberculosis* surveillance: a standardized, portable, and expandable approach. *J. Clin. Microbiol.* 52 2479–2486. 10.1128/jcm.00567-14 24789177 PMC4097744

[B8] Lee Y. Kim B. S. Chun J. Yong J. H. Lee Y. S. Yoo J. S. (2014). Clonality and Resistome analysis of KPC-producing *Klebsiella pneumoniae* strain isolated in Korea using whole genome sequencing. *BioMed Res. Int.* 2014:352862. 10.1155/2014/352862 PMC4106114 25105122

[B9] Magalhães B. Valot B. Abdelbary M. M. H. Prod’hom G. Greub G. Senn L. (2020). Combining standard molecular typing and whole genome sequencing to investigate *Pseudomonas aeruginosa* epidemiology in Intensive Care Units. *Front. Public Health* 8:3. 10.3389/fpubh.2020.00003 32047733 PMC6997133

[B10] Maiden M. C. van Rensburg M. J. J. Bray J. E. Earle S. G. Ford S. A. Jolley K. A. (2013). MLST revisited: the gene-by-gene approach to bacterial genomics. *Nat. Rev. Microbiol.* 11 728–736. 10.1038/nrmicro3093 23979428 PMC3980634

[B11] Mellmann A. Bletz S. Boking T. Kipp F. Becker K. Schultes A. (2016). Real-time genome sequencing of resistant bacteria provides precision infection control in an institutional setting. *J. Clin. Microbiol.* 54 2874–2881. 10.1128/jcm.00790-16 27558178 PMC5121374

[B12] Morrison A. J. Wenzel R. P. (1984). Epidemiology of Infections Due to *Pseudomonas aeruginosa*. *Rev. Infect. Dis.* 6 S627–S642. 6443765 10.1093/clinids/6.supplement_3.s627

[B13] Parcell B. J. Oravcova K. Pinheiro M. Holden M. T. G. Phillips G. Turton J. F. (2018). *Pseudomonas aeruginosa* intensive care unit outbreak: winnowing of transmissions with molecular and genomic typing. *J. Hosp. Infect.* 98 282–288. 10.1016/j.jhin.2017.12.005 29229490 PMC5840502

[B14] Pearce M. E. Alikhan N. F. Dallman T. J. Zhou Z. Grant K. Maiden M. C. J. (2018). Comparative analysis of core genome MLST and SNP typing within a European *Salmonella serovar* Enteritidis outbreak. *Int. J. Food Microbiol.* 274 1–11. 10.1016/j.ijfoodmicro.2018.02.023 29574242 PMC5899760

[B15] Perez-Vazquez M. Sola-Campoy P. J. Zurita A. M. Avila A. Gomez-Bertomeu F. Solis S. (2020). Carbapenemase-producing *Pseudomonas aeruginosa* in Spain: interregional dissemination of the high-risk clones ST175 and ST244 carrying *bla*_VIM–2_, *bla*_VIM–1_, *bla*_IMP–8_, *bla*_VIM–20_ and *bla*_*KPC–2*_. *Int. J. Antimicrob. Agents* 106026. 10.1016/j.ijantimicag.2020.106026 32450200

[B16] Pightling A. W. Petronella N. Pagotto F. (2014). Choice of reference sequence and assembler for alignment of *Listeria monocytogenes* short-read sequence data greatly influences rates of error in SNP analyses. *PLoS One* 9:e104579. 10.1371/journal.pone.0104579 25144537 PMC4140716

[B17] Royer G. Fourreau F. Boulanger B. Mercier-Darty M. Ducellier D. Cizeau F. (2020). Local outbreak of extended-spectrum beta-lactamase SHV2a-producing *Pseudomonas aeruginosa* reveals the emergence of a new specific sub-lineage of the international ST235 high-risk clone. *J. Hosp. Infect.* 104 33–39. 10.1016/j.jhin.2019.07.014 31369808

[B18] Stanton R. A. McAllister G. Daniels J. B. Breaker E. Vlachos N. Gable P. (2020). Development and application of a core genome multilocus sequence typing scheme for the healthcare-associated pathogen *Pseudomonas aeruginosa*. *J. Clin. Microbiol.* 10.1128/JCM.00214-20 [Epub ahead of print]. 32493782 PMC7448633

[B19] Struelens M. J. (1996). Consensus guidelines for appropriate use and evaluation of microbial epidemiologic typing systems. *Clin. Microbiol. Infect.* 2 2–11. 10.1111/j.1469-0691.1996.tb00193.x 11866804

[B20] Tissot F. Blanc D. S. Basset P. Zanetti G. Berger M. M. Que Y. A. (2016). New genotyping method discovers sustained nosocomial *Pseudomonas aeruginosa* outbreak in an intensive care burn unit. *J. Hosp. Infect.* 94 2–7. 10.1016/j.jhin.2016.05.011 27451039

[B21] van Belkum A. Tassios P. T. Dijkshoorn L. Haeggman S. Cookson B. Fry N. K. (2007). Guidelines for the validation and application of typing methods for use in bacterial epidemiology. *Clin. Microbiol. Infect* 13 1–46. 10.1111/j.1469-0691.2007.01786.x 17716294

[B22] Vincent J. L. Bihari D. J. Suter P. M. Bruining H. A. White J. Nicolas-Chanoin M. H. (1995). The prevalence of nosocomial infection in intensive care units in Europe. Results of the European Prevalence of Infection in Intensive Care (EPIC) Study. EPIC International Advisory Committee. *JAMA* 274 639–644. 10.1001/jama.274.8.639 7637145

