# Supplementary material for: Comparison of Whole Genome (wg-) and Core Genome (cg-) MLST (BioNumericsTM) Versus SNP Variant Calling for Epidemiological Investigation of Pseudomonas aeruginosa
Source: Front Microbiol. 2020 Jul 22;11:1729. doi: 10.3389/fmicb.2020.01729 (PMC7387498; doi:10.3389/fmicb.2020.01729)
Supplement: FIGURE S1 — SNP and wgMLST similarity trees of ST1076 isolates. [file Data_Sheet_1.PDF]

Supplementary Material

Comparison of wgMLST (BioNumerics™) versus SNP variant calling for epidemiological investigation of *Pseudomonas aeruginosa*

Dominique S. Blanc, Bárbara Magalhães, Isabelle Koenig, Laurence Senn and Bruno Grandbastien

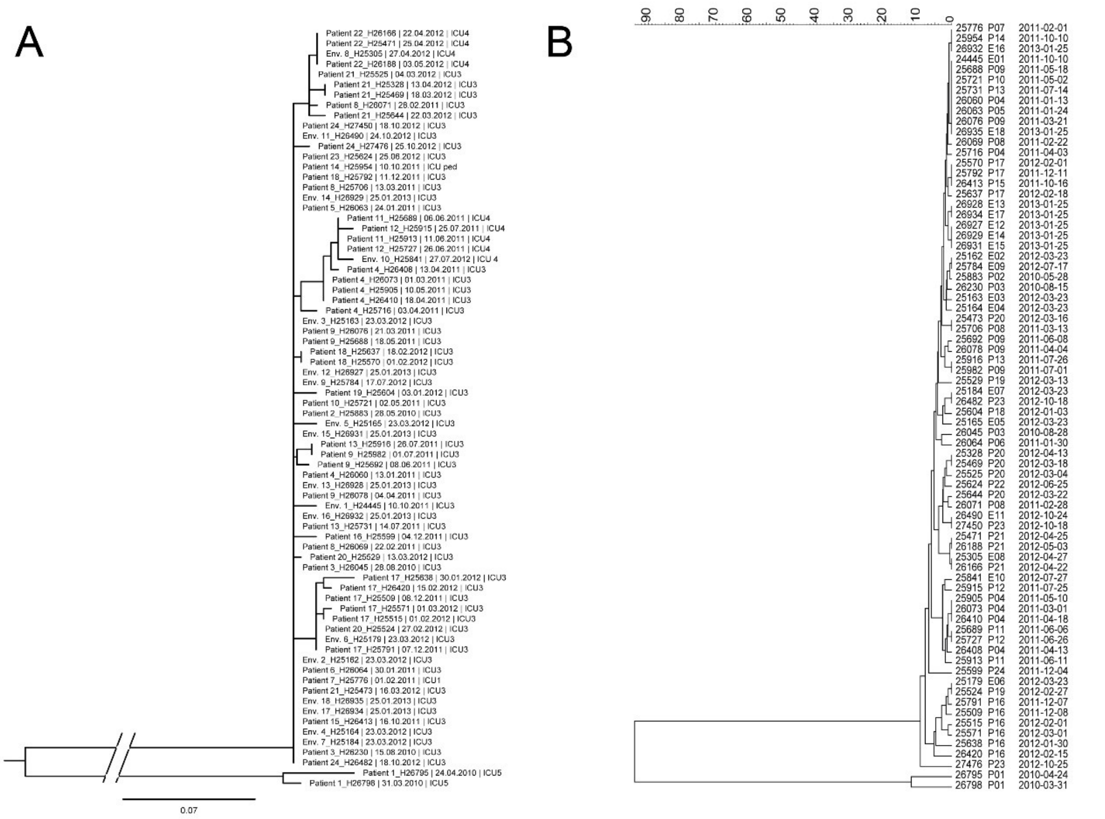

Figure S1. Phylogenetic trees of ST1076 isolates. **A.** Maximum likelihood tree based on SNP variant calling. **B.** UPGMA tree based on categorical similarity coefficient of wgMLST data; one unit represents one locus difference. The two isolates belonging to Patient 1 clustered apart from the remaining isolates.

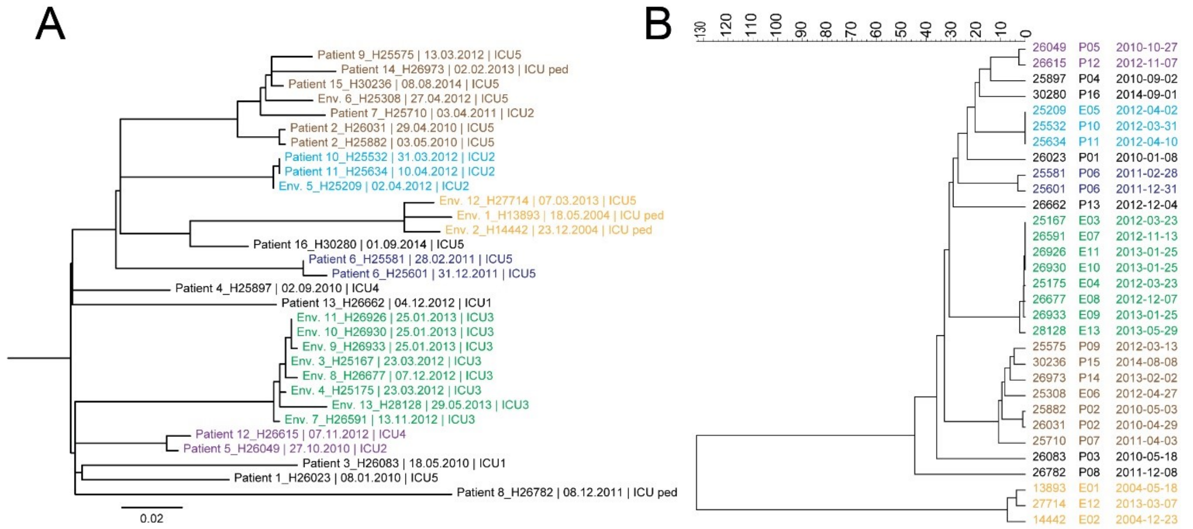

Figure S2. Phylogenetic trees of ST253 isolates. **A.** Maximum likelihood tree base on SNP variant calling. **B.** UPGMA tree based on categorical similarity coefficient of wgMLST data; one unit = one locus difference. Clusters of highly similar isolates are highlighted with the same colour in both trees. Epidemiological links were identified between patients #10, 11 and environment #5 (in blue); as well as between patients #5 and 12 (in purple).

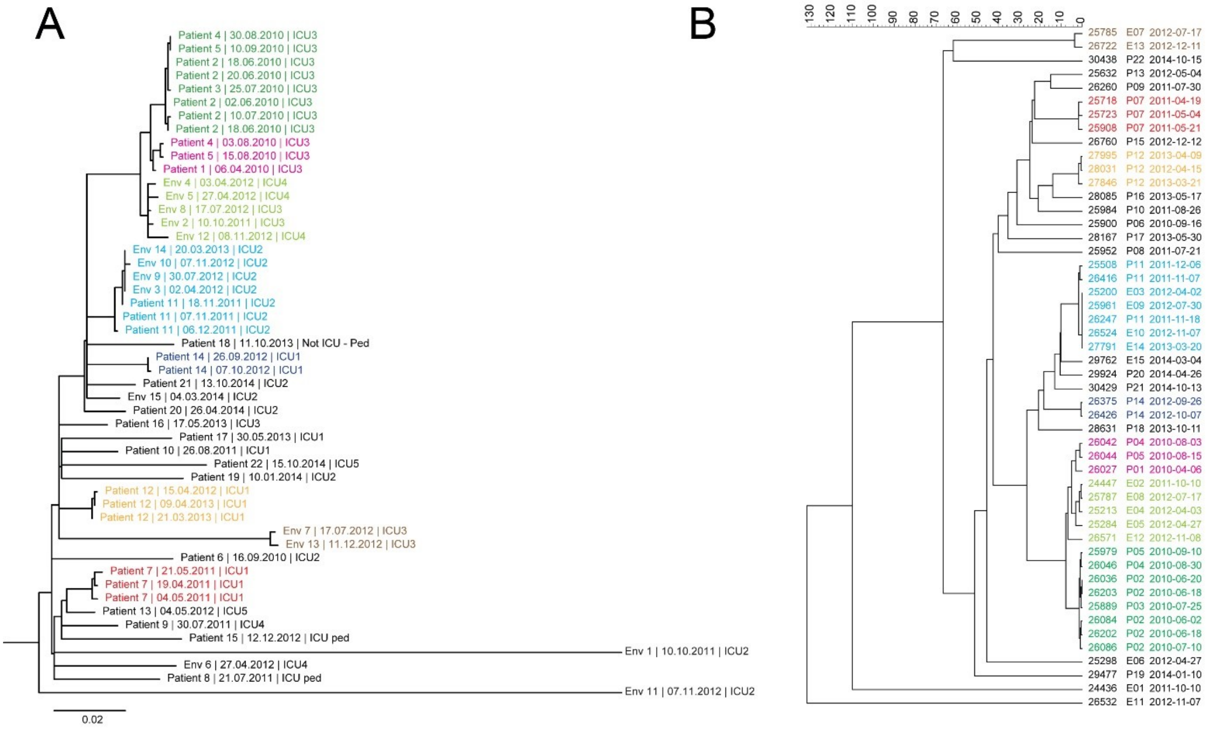

Figure S3. Phylogenetic trees of ST17 isolates. **A.** Maximum likelihood tree base on SNP variant calling. **B.** UPGMA tree based on categorical similarity coefficient of wgMLST data. One unit = one locus difference. Clusters of highly similar isolates are highlighted with the same colour in both trees. Epidemiological links were identified between patients # 1, 2, 3, 4 and 5 in green).

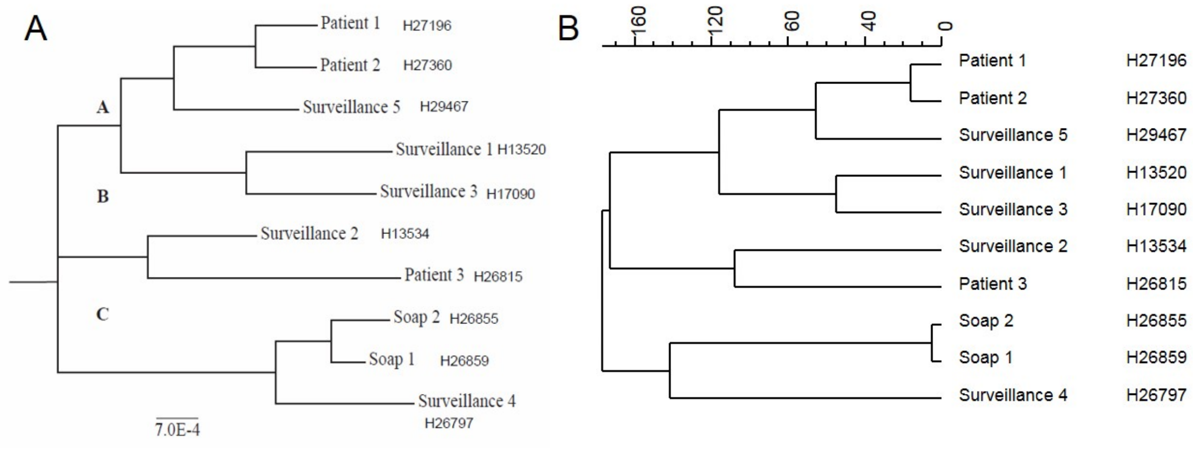

Figure S4. Phylogenetic trees of ST155 isolates. **A**. Maximum-likelihood phylogenetic tree based on the core SNP variant calling. **B.** UPGMA tree based on categorical similarity coefficient of wgMLST data. Patients 1 to 3 correspond to isolates of the three patients included in the study; soap isolates 1 and 2 indicate the isolates retrieved from two contaminated soap batches; and surveillance 1 to 5 represent clinical isolates recovered during routine epidemiological surveillance outside the study period.

Table S5. Number of variable loci and total number of loci identified in the four analysed dataset with wgMLST and cgMLST. The number of allele differences between outbreak isolates were also reported for the two schemes.

MLST- ST	wgMLST		cgMLST		Loss of variable loci
	variable loci	Allele differences in outbreak		Allele differences in outbreak	
ST1076	153/6066	0-12	150/5940	0-12	2%
ST17	1114/6678	0-17	838/5932	0-17	25%
ST253	667/7077	0	545/5797	0	18%
ST155	656/6920	5	407/5755	5	38%

Step by step procedure to performed a core genome MLST on a set of isolates in Bionumeric version 7.6.3

Perform a comparison with the isolates of the set

Select the experiment wgMLST

Select all loci (right click on the loci names)

Select the menu Statistics > Core locus analysis

Set the number of repeats to 100 (default)

Set the presence threshold to 90.0% (default)

Close the Chart and statistics windows

In the comparison windows, loci from the core genome are selected

Under the experiment wgMLST, select <selected characters>
